# Sociodemographic Predictors of Depression in US Rural Communities During COVID-19: Implications for Improving Mental Healthcare Access to Increase Disaster Preparedness

**DOI:** 10.1017/dmp.2022.203

**Published:** 2022-08-04

**Authors:** Clare EB Cannon, Regardt Ferreira, Fredrick Buttell, Chase Anderson

**Affiliations:** 1 University of California, Davis, CA, USA; 2 University of the Free State, Bloemfontein, South Africa; 3 Tulane University School of Social Work, New Orleans, LA, USA

**Keywords:** mental health, depression, PHQ-9, rural communities, disasters

## Abstract

**Objective:**

The objective of this research is to identify sociodemographic predictors of depression for a rural population in the US during the COVID-19 pandemic to enhance mental health disaster preparedness.

**Methods:**

This study uses t-tests to differentiate between gender and ethnicity groups regarding depression status; binary logistic regression to identify socio-demographic characteristics that predict depression status; and t-test to differentiate between average depression scores, measured by the PHQ-9, pre-COVID-19 pandemic (2019) and after it’s start (2020).

**Results:**

Results indicate that men were less likely than women to report depression. Clients who identified as Latinx/Hispanic were 2.8 times more likely than non-Hispanics to report depression and clients who did not reside in public housing were 19.9% less likely to report depression. There was a statistically significant difference between mean PHQ-9 scores pre- and post-pandemic, with pre-pandemic scores lower on average, with a small effect size.

**Conclusions:**

Building on findings from this study, we propose ways to increase rural access to mental health services, through equitable access to telemedicine, to meet the needs of rural clients to increase disaster preparedness.

Like other kinds of disasters, the coronavirus disease 2019 (COVID-19) pandemic, as an infectious disease disaster, has likely increased rates of depression across the US.^1^ Even prior to this pandemic, 1 in 6 U adult residents were estimated to have experienced depression in their lifetime. Experiences of depression can be particularly devastating, as they likely increase comorbidities such as anxiety, pain, and cardiovascular disease.^2^

Few studies have investigated relationships between the COVID-19 pandemic and depression in US rural communities.^3^ Prior disaster-focused research has looked at relationships among increased depression after disaster and associated factors such as resource loss, suicidal ideation, and social support in rural areas globally. Yet, little is known of the role of demographic factors in influencing experiences of depression during the COVID-19 pandemic in rural communities in the US.

Generally, it is unclear whether there are higher rates of depression in rural areas in the US compared to urban areas – with some research finding depression rates are slightly higher in rural areas.^4^ What is known is that roughly 20% of Americans live in rural areas and 20% of that population have a mental illness.^5^ Yet it is not well understood whether the prevalence of depression varies by gender, race, or ethnicity in rural communities, with research suggesting, generally, that women are more likely than men to experience depression, and to what extent comorbid effects such as higher rates of poverty, may contribute to rates of depression.^4^ Regardless, people living in US rural communities experience a resource gap in medical services and it is clear that depression in these areas is chronically understudied. 2 reasons behind rural mental health disparities include poor access to health care and limited availability of mental health providers.^3^ Such lack of resources is particularly acute during disasters, laying the groundwork for exacerbating already existing mental health disparities for rural residents. Given the lack of resources in rural areas, person centered interventions are essential to reducing experiences of depression and their comorbid effects, all while increasing disaster preparedness.^6^

To address this gap in knowledge, the current study investigated sociodemographic predictors of depression among a sample of clients who requested services from a Federally Qualified Health Center (FQHC) in 2019 and 2020. Federally Qualified Health Centers (FQHC) are community-based health centers that provide health care services in areas with limited resources. FQHC have stringent requirements they must meet to qualify for funding from the Health Resources and Services Administration. They must also provide services on a sliding scale, which is tied to household income and/or a patient’s lack of insurance.

## Methods

This study utilized de-identified data from FQHC clinics serving contiguous rural communities that span North-East Texas, North-West Louisiana, and South-West Arkansas. The Tulane Social/Behavioral Institutional Review Board approved this study. Data consisted of all documented visits of individuals throughout 2019 and 2020 for which data were available (n = 2762). Specifically, this study used the PHQ-9, a well-validated measure for assessing depression,^7^and demographic variables (e.g., race, ethnicity) to advance understanding of how the COVID-19 pandemic intersects with demographic variables for a rural population. The PHQ-9 was administered to clients and demographic measures collected as part of the intake process for the FQHC. Analysis included both t-tests and a series of binary logistic regression models that focused on both average rates of depression among the rural sample and investigations into differences in depression by race, ethnicity, and gender. Another t-test was used to identify differences in pre-COVID-19 pandemic mean PHQ-9 scores (2019) and mean PHQ-9 scores after the pandemic’s start (2020).

## Results

The sample of 2762 clients had a mean age of 43.87 years (SD = 20.42), with 62.0% identifying as females (n = 1713), 33.8% as men (n = 934), and 4.2% (n = 115) as other. Most of the sample identified as white 79.6% (n = 2199). Regarding ethnicity, 3.1% (n = 87) identified as Hispanic/Latino. Less than 1% (0.7%, n = 18) identified English as a second spoken language. With respect to veteran status, 2.8% (n = 78) of the sample identified as being veterans. Regarding public housing 1.1% (n = 30) of the sample were making use of public housing. Most of the sample (99.9%, n = 2759) did not identify as immigrants. The sample had 0.2% (n = 5) respondents that reported homelessness. Regarding the PHQ-9, respondents had a mean score of 9.48 (SD = 6.71) for the 9-item questionnaire. Table 1 provides a detailed description of the sample.

**Table 1.** Demographic characteristics

	Clients (n = 2762)		
Characteristic (coded value)	Mean/%	n Range	SD
Age (in years)	43.87	744	15.35
		18 - 91	
Gender			
Female	62.0	1713	
Male	33.8	934	
Other	4.2	115	
Race			
White	79.6	2199	
Black	17.2	476	
Native American/Alaska Native	0.5	16	
Asian	0.4	11	
Other	2.3	60	
Ethnicity			
Hispanic/Latino	3.1	87	
Non-Hispanic/Latino	93.9	2594	
Did not disclose	2.9	81	
English Second Language			
Yes	0.7	18	
No	99.3	2744	
Veteran Status			
Yes (1)	2.8	78	
No (2)	97.2	2684	
Public Housing			
Yes (1)	1.1	30	
No (2)	98.9	2732	
Migrant Status			
Yes (1)	0.1	3	
No (2)	99.9	2759	
Homeless Status			
Yes (1)	0.2	5	
No (2)	99.8	2757	
Patient Health Questionnaire 9 (PHQ-9) Mean Score	9.48		6.71
PHQ-9 Binary Category (score)			
None or low depression (0 - 9)	55.6	1535	
Depression (10 - 27)	44.4	1227	
PHQ-9 Mean Score Pre-pandemic (2019)	6.65	2438	.14
PHQ-9 Mean Score Post-pandemic (2020)	7.09	324	.39

### Group differences for depression score (PHQ-9) for gender and race

An independent sample t-test was performed to determine if there was a difference between mean scores of PHQ-9 experienced by males and females. The mean PHQ-9 score for the male group was 8.25 (SD = 6.38) and for the female group was 10.12 (SD = 6.79), indicating that the female group reported higher levels of depression compared to the male group. This difference was significant (t (2645) = 6.905, *P = 0.001*) with a small effect size (Cohen’s d = 0.283). An independent sample t-test was performed to determine if there was a difference between mean scores of depression experienced by 2 racial groups, white/minority, on the PHQ-9. The mean PHQ-9 score for the white group was 9.61 (SD = 6.82) and for the minority group was 9.00 (SD = 6.25), indicating that the white group reported higher levels of depression compared to the minority group. This difference was significant (t (2760) = 1.935, *P = 0.027*) with a small effect size (Cohen’s d = 0.091).

### Model testing: predicting group membership for depression and gender

2 logistic regression models investigated group membership based on depression and gender with their odds ratios presented in Table 2. A binary logistic regression analysis was performed to investigate how well age, gender, race, ethnicity, veteran status, and public housing status predicted group membership based on depression status. To create a binary depression variable, PHQ-9 scores of 0 - 9 were used to create the category termed ‘none or low depression’ and PHQ-9 scores of 10 - 27 were used to create the category termed ‘depression.’^7^ The group that did not exhibit depression was 55.6% (n = 1535) of the sample compared to 44.4% (n = 1227) of the sample that did exhibit depression. The model consisted of 6 predictors and allowed for simultaneous entry of all the independent variables. A test of the full model against a constant-only model was statistically significant (*χ* ^2^ = 21.84, df = 6, *P = 0.01*). Prediction success for the cases used in the development of the model had an overall success rate of 94.1%. Gender, ethnicity status, and public housing status were statistically significant. Women were 1.3 times more likely than men to belong to the depression group. Clients who identified as Latino/Hispanic were 2.8 times more likely than non-Hispanics to experience depression. Clients who did not reside in public housing were 19.9% less likely to experience depression.

**Table 2.** Logistic regression models predicting membership in depression group, gender group, and racial group

Logistic regression predicting depression group membership (n = 2749; df = 6)							
						95% CI for Exp(B)	
	B	S.E.	Wald	Exp (B)	Sig.	Lower Bound	Upper Bound
Age (in years)	0.001	0.004	0.020	1.001	0.889	0.993	1.009
Gender**	0.291	0.099	8.701	1.338	0.003	1.103	1.624
Race	0.098	0.199	0.244	1.103	0.621	0.747	1.630
Ethnicity***	1.042	0.297	12.273	2.834	0.001	1.582	5.075
Veteran Status	−0.551	0.390	1.989	0.577	0.158	0.268	1.239
Public Housing**	−0.222	0.742	0.089	0.801	0.765	0.187	3.427
Constant	−3.956	1.804	4.810	0.019	0.019		

Logistic regression predicting gender group membership (n = 2681; df = 6)							
						95% C.I for EXP (B)	
	B	S.E.	Wald	Exp (B)	Sig.	Lower	Upper
Age (in years)**	0.006	0.002	8.232	1.006	0.004	1.002	1.010
Race	−0.061	0.100	0.375	0.941	0.541	0.774	1.144
Ethnicity	−0.033	0.162	0.041	0.978	0.840	0.705	1.329
Veteran Status***	1.954	0.280	48.697	7.054	0.001	4.075	12.210
Public Housing	−0.867	0.462	3.518	0.420	0.061	0.170	1.040
PHQ9***	−0.650	0.165	15.451	0.522	0.001	0.378	0.722
Constant	−1.643	1.141	2.076	0.193	0.150		

**P < 0.01, *** P < 0.001

A second model assessed how well age, race, ethnicity, veteran status, public housing status, and PHQ-9 predicted group membership based on gender. The model consisted of 6 predictors and allowed for simultaneous entry of all the independent variables. A test of the full model against a constant-only model was statistically significant (*χ* ^2^ = 86.22, df = 6, *P = 0.001*). Prediction success for the cases used in the development of the model were moderate, with an overall success rate of 65.1%. Age, veteran status, and depression status were statistically significant. Older clients were more likely to belong to the female group as were non-veterans compared to veterans. Clients experiencing depression were also 47.8% more likely to be women.

### Group differences for depression score (PHQ-9) for pre-COVID-19 pandemic and after the start of the pandemic

There were 2438 individuals for whom data was available pre-COVID-19 pandemic (pre-covid, 2019) and 324 with data available after the start of the COVID-19 pandemic (post-Covid, 2020). An independent t-test was performed to determine if there were differences in PHQ-9 scores before and after the start of the COVID-19 pandemic in the US. The average PHQ-9 score post-covid (mean = 7.09, SD = 0.39) was higher than pre-covid scores (mean = 6.65, SD = 0.14). There was a statistically significant difference between the 2 groups (t(402.32) = -6.25; *P < 0.001*). The pre-covid PHQ-9 mean score was 0.44 (SE = 0.416) lower than the average post-covid PHQ-9 score, with a small effect size (95% CI = -0.342, -1.78: Cohen’s *d* = 0.39).

## Limitations

There are limitations to the current research. The data is cross-sectional and cannot point towards direct causality between the explanatory and outcome variables. The data represent those that visited a FQHC, limiting the generalizability of the research. Although data from FQHC are an important step in identifying relationships between socio-demographic variables and depression, the sample was overwhelmingly white, limiting the ability of the statistical tests to determine key factors in the relationship between race and depression. Additional data such as drive time and residential zip codes would be useful in assessing accessibility of such centers. Future research should use data more inclusive of racial/ethnic differences and to assess accessibility. Future research should also consider identifying key similarities and differences between rural and urban populations and depression.

## Discussion

The research presented here is some of the first to empirically assess potential linkages among depression, race, and gender to an infectious disease disaster for a rural sample. The findings suggest that women, people living in public housing (a strong indicator of poverty), and clients who identified as Latinx/Hispanic were more likely to report depression. The finding that women self-report more depression than men is consistent with data more generally, as is the finding that Hispanic/Latinx clients reported more depression than non-Hispanic/Latinx clients, which may be related to the marginalization of these clients based on ethnicity.^8^ Importantly, rural women, older clients, and clients who identified as Latino/Hispanic were more likely to have experienced depression during the COVID pandemic, relative to male clients, younger clients, and those who identified as white. Finally, like other research into the COVID-19 pandemic,^1^ average depression scores measured by the PHQ-9 were higher after the start of the pandemic than before for this sample of rural US residents, evidencing the urgent need to further develop disaster preparedness interventions for mental health, particularly for rural residents, who frequently experience a resource gap in health services. Although this is just 1 study, the findings shed some light on rural people’s experiences with the COVID-19 pandemic and may help inform ways to assist these populations in preparing for the next infectious disease disaster. For example, these findings were provided to the agency partner to assist them in refining their programming in order to increase access to mental health services for rural clients, train health care providers to ask screening questions for all clients including people from diverse racial and ethnic backgrounds, craft person-centered interventions that focus on client strengths and capacities, and increase public health preparedness to disaster.^6^

Though there is a solid body of research supporting the use of tele-health for addressing mental health issues,^9^ this is not an immediate solution for improving rural residents’ access to mental health services and disaster preparedness. Rural areas suffer from a digital divide relative to urban areas and recent research suggests that as many as 42 million Americans do not have access to broadband.^10^ Although the CARES Act (2021) set aside money to improve broadband access in rural areas, that will take time. In the meantime, asking depression screening questions of all clients who request services is achievable and makes sense, given these findings. More difficult, but longer-term strategies, include sending out mobile units to see people in rural areas by making use of public spaces, such as schools and other easily accessed public areas (e.g., libraries). As noted above, these efforts should focus on crafting personalized interventions that meet the needs of the rural client to increase disaster preparedness.

## Conclusion

The COVID-19 pandemic continues to impact individuals, their families, and communities. Such impacts for rural communities have not been well understood since the pandemic started in the US in 2020. Building on findings from this study, we propose ways to increase rural access to mental health services, through equitable access to telemedicine, as well as suggest personalized interventions that meet the needs of the rural client to increase disaster preparedness.
